# Reduction Study of Carbon-Bearing Briquettes in the System of Multiple Reductants

**DOI:** 10.3390/ma18184408

**Published:** 2025-09-21

**Authors:** Xiaojun Ning, Zheng Ren, Nan Zhang, Guangwei Wang, Xueting Zhang, Junyi Wu, Jiangbin Liu, Andrey Karasev, Chuan Wang

**Affiliations:** 1 School of Metallurgical and Ecological Engineering, University of Science and Technology Beijing, Beijing 100083, China; 2 Henan Iron and Steel Group, Henan Advanced Steel Materials Industry Research Institute, Zhengzhou 450046, China; 3 Material Science and Engineering, KTH Royal Institute of Technology, SE-10044 Stockholm, Sweden

**Keywords:** wood char, bamboo char, carbon-bearing briquettes, gasification characteristics, metallization rate, microstructure

## Abstract

Against the backdrop of escalating global carbon emissions, the steel industry urgently requires a transition toward green and low-carbon practices. As a conditionally carbon-neutral renewable energy source, biochar holds potential for replacing traditional fossil-based reducing agents. This study aims to investigate the mechanism and performance differences between biochar (wood char, bamboo char) and conventional reducing agents (semi-coke, coke powder, anthracite) in the direct reduction process of carbon-bearing briquettes. Through reduction experiments simulating rotary kiln conditions, combined with analysis of reducing agent gasification characteristics, carbon-to-oxygen (C/O) molar ratio control, X-ray diffraction (XRD), and microstructural examination, the high-temperature behavior of different reducing agents was systematically evaluated. Results indicate that biochar exhibits superior gasification reactivity due to its high specific surface area and developed pore structure: wood char and bamboo char show significantly enhanced reaction rates above 1073 K, approaching complete conversion at 1173 K. In contrast, anthracite and coke powder, characterized by dense structures and low specific surface areas, failed to achieve complete gasification even at 1273 K. Pellets containing bamboo char achieved the highest metallization rate (90.16%) after calcination at 1373 K. The compressive strength of the pellets first decreased and then increased with rising temperature, consistent with the trend in metallization rate. The mechanism analysis indicates that the high reactivity and porous structure of biochar promote rapid CO diffusion and synergistic gas–solid reactions, significantly accelerating the reduction of iron oxides and the formation of metallic iron.

## 1. Introduction

As global carbon dioxide emissions and the greenhouse effect continue to intensify, climate change has emerged as a pressing global issue. To reduce carbon emissions and guide the steel industry toward the goals of “carbon peaking” and “carbon neutrality,” a transition to green, low-carbon metallurgical processes is imperative [1,2,3]. As a renewable energy source with conditional carbon neutrality characteristics, biomass offers a promising alternative to traditional fossil fuels for steel production. This is primarily due to its fundamentally different carbon cycle process compared to fossil fuels: biomass belongs to a fast carbon cycle, absorbing atmospheric CO_2_ through photosynthesis during growth. When used in industrial production (such as preparing biochar or reducing iron oxides), the released CO_2_ essentially returns to its original cycle without adding “new” carbon to the atmosphere. In contrast, fossil coal (such as anthracite and coke powder) belongs to the slow carbon cycle. Its carbon has been sequestered underground for millions of years, so its combustion releases carbon that has been removed from the atmospheric cycle for extended periods, directly contributing to rising atmospheric CO_2_ concentrations. However, this “carbon neutrality potential” of biomass is not absolute. Its realization depends on a crucial prerequisite: the biomass consumed in industrial processes must be replenished by planting an equivalent amount of fast-growing tree species or other plants, thereby maintaining the balance of the fast carbon cycle [4,5,6]. Only under this condition can the substitution of fossil carbon with biomass carbon in reduction processes be considered “conditional zero-carbon.”

It is particularly important to note that the carbon footprint of biochar (a key biomass-derived material used in this study) also includes emissions from its production process. Whether it is the thermal energy required for pyrolysis or the electricity needed for drying, if these energy sources come from fossil fuels (such as coal-fired heating or grid electricity dominated by thermal power), additional carbon emissions will be generated, potentially partially offsetting their low-carbon advantage. Existing research indicates that the carbon intensity of biochar production is closely tied to energy sources: for instance, utilizing pyrolysis by-products like pyrolysis gas for self-heating, or pairing with renewable energy like solar or wind power, can reduce preparation-stage emissions by 30–50% compared to fossil fuels [7,8,9].

The biomass-derived biochar used in this study (e.g., wood char, bamboo char) inherits the aforementioned conditional carbon advantages while also possessing high reactivity and abundant pore structures [10]. When applied in steel production, under the premise of ensuring biomass replanting and low-carbon preparation, it not only helps reduce net carbon emissions but also decreases the release of sulfur oxides and nitrogen oxides [11,12]. In the blast furnace injection field, biochar has been partially substituted for coal powder to achieve carbon emission reduction [13,14,15]. In the sintering process, its high reactivity has been utilized to optimize fuel combustion efficiency [16,17,18,19]. In the coking process, attempts have been made to co-carbonize with coal to improve the microstructure of coke [20,21,22]. Particularly in the field of carbon-bearing briquettes, biochar, with its high reactivity, abundant pore structure, and renewable characteristics, has become a research hotspot when used as a new reducing agent to produce carbon-bearing briquettes with iron ore powder.

In recent years, scholars have conducted extensive research on the behavior of biochar in the reduction of carbon-bearing briquettes. Pouria [23] replaced coke with rice husk charcoal to reduce carbon-bearing briquettes at 1200 °C, achieving a metallization rate of 88% and an 18% reduction in CO_2_ emissions. And the high reactivity of biochar shortened the reduction time. Dipika [24] applied biomass as a reducing agent in iron ore reduction, enhancing the carbon-neutral properties of biomass. Additionally, using corn cob volatiles to reduce iron ore briquettes was 56% cheaper than using coal. Fu [25] proposed that coconut shell charcoal and bamboo char possess excellent metallurgical properties, with crush strength values suitable for the RHF process. Zhao [26] proposed that the strength of biomass-containing carbon briquettes exhibits a trend of first decreasing and then increasing with rising reduction temperature. At 900–1000 °C, lattice distortion causes volume expansion, resulting in the lowest strength; after 1200 °C, pellet strength and metallization rate increase synchronously. Currently, metallurgical researchers have conducted extensive studies on the reduction of carbon-bearing briquettes using biochar. Extensive research on biochar reduction of carbon-bearing briquettes has been conducted, but there is still a lack of systematic comparative research with traditional fossil reducing agents such as coke powder and anthracite.

This study investigates the reduction performance of biochar reductants compared to conventional reductants in iron oxide briquettes at varying temperatures, as well as the underlying mechanism of iron oxide reduction by biochar. To this end, biochar (wood char, bamboo char), semi-coke, coke powder, and anthracite were used as reducing agents to reduce carbon-bearing briquettes. Reduction experiments were conducted under laboratory conditions that simulated the heating process and working temperature range of a rotary kiln. The reduction mechanism of biochar in carbon-bearing briquettes was investigated through comprehensive characterization of reductant gasification kinetics, carbon-to-oxygen (C/O) molar ratios, briquette compressive strength, and metallization rates across varying temperatures. This experimental framework, integrating X-ray diffraction (XRD) analysis with microstructural characterization, elucidated biochar’s role in direct reduction processes.

## 2. Materials and Methods

### 2.1. Raw Material Properties

**Figure 1:**
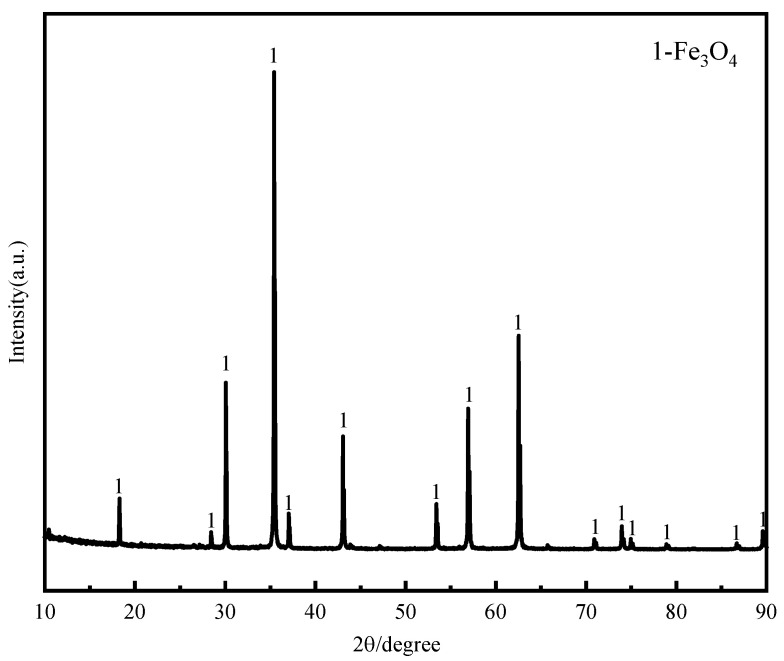
XRD pattern of iron concentrate.

The iron concentrate used in the experiment was sourced from a major domestic steel company. XRF and XRD analyses were conducted on the iron concentrate. The XRF analysis results are shown in Table 1. As can be seen, the iron concentrate has a TFe content of 70.84% and an FeO content of 22.76%, and the gangue primarily consists of quartz (SiO_2_) with a content of 1.18%, MgO with a content of 1.01%, and relatively low Al_2_O_3_ and CaO contents, with values of 0.58% and 0.36%, respectively. The XRD results are shown in Figure 1, indicating that the primary mineral component of the iron concentrate is magnetite.

The reducing agents used in the preparation of carbon-bearing briquettes include wood char, bamboo char, semi-coke, anthracite, and coke powder. Based on GB/T 212-2001 and GB/T 476-2001 standards, industrial analysis and elemental analysis were performed on the reducing agents. The results are shown in Table 2. The composition of wood char and bamboo char is close to that of anthracite. Bamboo char has the highest fixed carbon content and the lowest ash content, reaching 90.16% and 2.76%, respectively.

### 2.2. Sample Preparation and Characterization

Iron concentrate powder was dried at 378 K for 12 h in a convection oven. Subsequently, reductants underwent identical drying conditions (378 K, 12 h), followed by comminution and sieving to isolate particles ≤0.150 mm. The carbon-to-oxygen (C/O) molar ratio was regulated through precise mass control of iron ore concentrate and reductant. A stoichiometrically determined binder quantity was uniformly blended into the mixture, followed by incremental addition of deionized water to achieve optimal pelletization consistency. The mixture is thoroughly mixed and allowed to stand for 10 min. Cylindrical briquettes (Ø25 mm × 20 mm height) were then compacted under 20 MPa uniaxial pressure using a hydraulic briquette press. After drying, the briquette samples were placed in an alumina crucible and transferred into a muffle furnace for calcination under specified process parameters. Upon completion of the calcination process, nitrogen (N_2_) was introduced into the furnace to isolate the samples from air, allowing them to cool to room temperature in an inert atmosphere.

In this study, metallic iron and total iron were detected using an automatic potentiometric titrator, and the metallization rate of the sample was calculated. The metallization rate (M) is an important indicator of the reduction efficiency of reactive briquettes, calculated using Equation (1):

(1) $$M=\frac{\mathrm{MFe}}{\mathrm{TFe}}\times 100\%$$

where M is the metallization rate, %; MFe is the metal iron content in carbon-bearing briquettes, %; TFe is the total iron content in carbon-bearing briquettes, %.

This study uses a thermogravimetric analyzer to detect the basic vaporization reactivity of reducing agents in order to characterize their vaporization properties at high temperatures. The instrument automatically collects data on weight loss and weight loss rates during the reaction process, and the weight loss data is used to calculate the vaporization conversion rates of different reducing agents. The conversion formula is shown in Equation (2):

(2) $$x=\frac{m_{0}-m_{t}}{m_{0}-m}$$

where *m*_0_ is the initial mass of the sample at time t_0_, g; *m_t_* is the mass of the sample at time t, g; *m* is the residual mass of the sample at the end of the reaction, g.

This study utilized a Zeiss EVO-18 scanning electron microscope (SEM) with an acceleration voltage of 20 kV, a working distance of 11.5 mm, and a magnification of 2.5 K. SEM images provided a more intuitive understanding of the surface microstructure of the reducing agent and carbon-bearing briquettes. An X-ray diffraction analyzer (XRD, Smart Lab, Okayama City, Japan) with a copper target (λ = 0.1541 nm) was used to determine the sample’s phase composition within a 2θ range of 10–90° at a scanning speed of 0.3°/min.

## 3. Results and Discussion

### 3.1. Gasification and Characteristics of Reducing Agents

**Figure 2:**
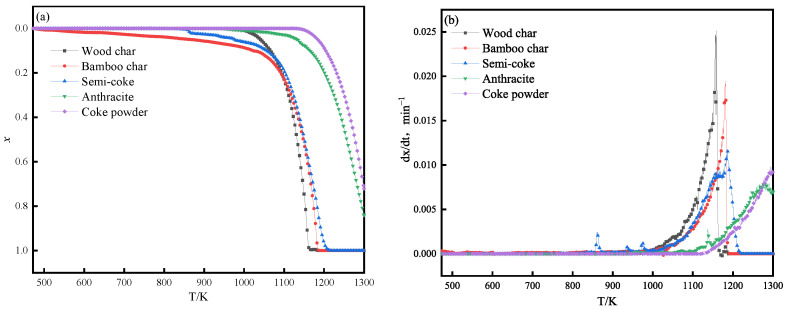
Conversion and reaction rate curves for different reducing agents: (**a**) conversion rate curve; (**b**) reaction rate curve.

Reductant gasification reactivity with CO_2_ was quantified via thermogravimetric analysis, with conversion and reaction rate profiles presented in Figure 2. Biochar exhibited significantly lower reaction onset temperature than anthracite and coke powder, demonstrating its enhanced gasification reactivity relative to conventional carbonaceous reductants.

As can be seen from the conversion rates of different reducing agents in Figure 2a, bamboo char began to lose weight first, followed by semi-coke and wood char, and finally anthracite and coke powder. When the temperature rose to 1073 K, the weight loss of the reducing agents changed significantly. At this point, the weight loss rate of wood char increased sharply, becoming the reducing agent with the most obvious increase in conversion rate, followed by bamboo char. The active performance of both at this stage further highlighted the high reactivity of biochar reducing agents. As the temperature continued to rise, when it reached about 1173 K, the weight loss rates of wood char, bamboo char, and semi-coke had already reached 100%, indicating that they had completely converted at this temperature node, and the material loss was exhausted. In contrast, when the temperature rose to 1273 K, the weight loss rates of anthracite and coke powder had not yet reached 100%, and a considerable portion of the material had not yet participated in the reaction. Therefore, it can be seen that the temperature range required for wood char, bamboo char, and semi-coke to lose weight is much lower than that required for anthracite and coke powder, once again proving the advantage of biochar as a reducing agent in terms of reactivity.

Figure 2b shows the reaction rate curves of different reducing agents. It can be seen from Figure 2b that before 1073 K, there is no obvious change in the reaction rate curve of the reducing agent. After 1073 K, the reaction rate of wood char is higher than that of bamboo char; the reaction rate of semi-coke is slightly lower than that of bamboo char, anthracite is second, and coke powder is the worst.

**Figure 3:**
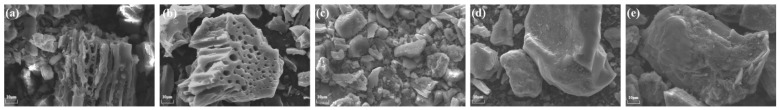
Original microstructure of different reducing agents: (**a**) wood char; (**b**) bamboo char; (**c**) semi-coke; (**d**) anthracite; (**e**) coke powder.

Scanning electron microscopy (SEM) analysis (Figure 3) reveals distinct microstructural characteristics governing reductant reactivity. Wood char and bamboo char exhibit highly porous carbon matrices with extensive specific surface areas, correlating with their enhanced gasification reactivity. This structural configuration facilitates rapid reaction initiation at lower temperatures. Conversely, anthracite and coke powder feature densely packed carbon architectures with limited surface development, resulting in diminished reactivity that necessitates elevated temperatures for significant gasification.

### 3.2. The Effect of the C/O

**Figure 4:**
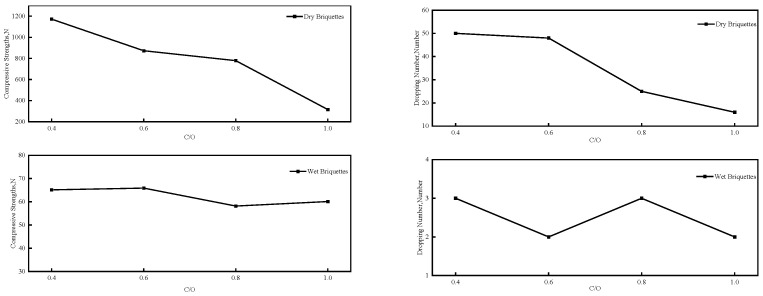
Comparison of different C/O ratios in carbon-bearing briquettes added to wood char.

The carbon-to-oxygen (C/O) molar ratio critically influences both direct reduced iron (DRI) productivity and process economics. Excessively high ratios induce reductant surplus, significantly increasing raw material costs while providing diminishing returns. Concurrently, sustained carbon diffusion through the metallic iron layer governs reaction kinetics. Therefore, selecting the appropriate C/O molar ratio is a necessary condition for achieving efficient reduction. The performance of carbon-bearing briquettes with different C/O molar ratios is shown in Figure 4. This study selected wood char as the reducing agent for preparing carbon-bearing briquettes due to its porous structure and moderate gasification reactivity, making it a representative biochar. Furthermore, adhering to the principle of single-variable experimentation, the optimal C/O ratio was determined to provide a unified benchmark for subsequent experiments involving other reducing agents.

While green briquette strength remains comparable across varying C/O molar ratios (Figure 4), dry briquette strength exhibits a significant inverse correlation with increasing carbon-to-oxygen stoichiometry. Despite compositional similarities between biochar and conventional carbon reductants (coal/coke powders), biochar exhibits substantially lower bulk density. Increasing the C/O molar ratio elevates the volumetric fraction of biochar, consequently compromising binder efficacy through reduced interparticle cohesion. When the C/O molar ratio is low, such as briquettes with C/O = 0.4 and C/O = 0.6, the dry briquette strength is 1172 N and 872.6 N, respectively. However, during the calcination self-reduction process, the insufficient carbon content in the carbon-bearing briquettes may hinder the reduction process, resulting in a low metallization rate of the calcination product. Conversely, when the C/O molar ratio is too high, such as briquettes with C/O = 1.0, the dry briquette strength is only 315.6 N. During the roasting process, due to the excessive amount of carbon added, the metallic iron phase connectivity of the carbon-bearing briquettes deteriorates, and the porosity and raw material costs increase, leading to a decrease in briquette strength. Considering both dry briquette strength and reduction efficiency, subsequent experiments selected briquettes with a C/O molar ratio of 0.8 for preparation.

### 3.3. The Effect of Different Reduction Temperatures

**Figure 5:**
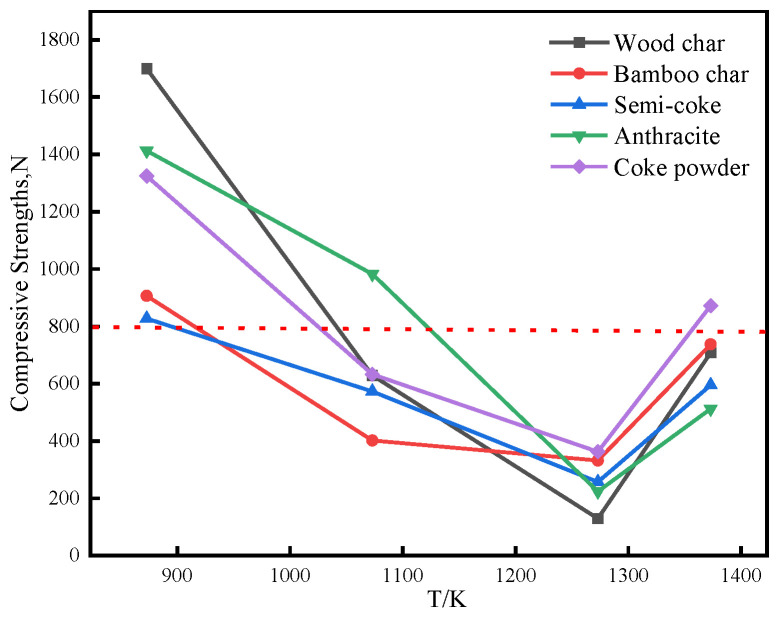
Effect of different reducing agents on the strength of carbon-bearing briquettes.

Carbon-bearing briquettes with a fixed C/O molar ratio of 0.8 were prepared using discrete reductants. These specimens underwent isothermal calcination at 873, 1073, 1273, and 1373 K for 30 min under an inert atmosphere to evaluate reductant-dependent performance variations. Figure 5 quantifies the corresponding compressive strength differentials induced by distinct carbon sources across the temperature spectrum.

The main criteria for measuring the strength of carbon-bearing briquettes are compressive strength and drop strength [27]. Following calcination at 873 K, activation of the composite binder yielded universally elevated compressive strengths (>800 N) across all reductant formulations. Briquettes incorporating wood char demonstrated exceptional mechanical integrity (1699 N), while semi-coke formulations constituted the control group with minimum strength (827 N). Upon thermal elevation to 1073 K, wood char-based briquettes exhibited a marked strength degradation of 58.3%, relative to their 873 K performance. This strength degradation primarily stems from wood char’s enhanced reactivity, which accelerates iron oxide reduction kinetics. Rapid phase transformation induces lattice strain and structural defects, thereby compromising briquette integrity. Notably, bamboo char-based briquettes exhibited minimal compressive strength (402 N), whereas anthracite formulations achieved peak mechanical performance (983 N) at this stage. Following calcination at 1273 K, all reductant systems manifested strength minima due to pervasive structural reorganization. Subsequent temperature elevation to 1373 K promoted extensive metallic iron formation, generating reinforcing iron whisker networks that reversed strength degradation trends.

**Figure 6:**
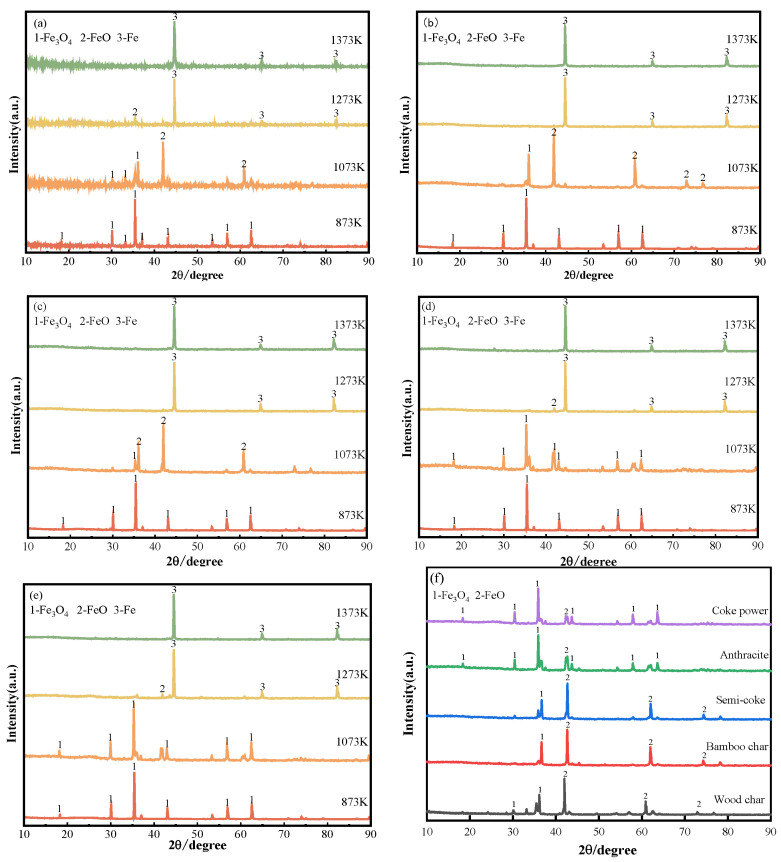
Phase composition of carbon-bearing briquettes after calcination with different reducing agent ratios: (**a**) wood char; (**b**) bamboo char; (**c**) semi-coke; (**d**) anthracite; (**e**) coke powder; (**f**) phase composition of different reducing agents at 1073 K.

Figure 6 delineates the phase evolution of carbon-bearing briquettes across reductant types and calcination temperatures. At 873 K, all systems remained unreduced with predominant Fe_3_O_4_ phases, indicating insufficient thermal energy for initiating iron oxide reduction. Progressive temperature elevation induced divergent reduction pathways among reductants, manifesting distinct phase transformation kinetics.

At 1073 K, briquettes utilizing coke powder or anthracite reductants maintained predominant Fe_3_O_4_ phase composition (Figure 6), indicating insufficient reaction kinetics for oxide reduction initiation. By contrast, wood char, semi-coke, and bamboo char systems exhibited partial transformation (Fe_3_O_4_→FeO) at equivalent temperature due to enhanced reduction kinetics. Progressive heating to 1273 K initiated metallic iron formation in highly reactive reductant systems (complete Fe phase in wood/bamboo char), while coke/anthracite formulations commenced primary reduction (Fe_3_O_4_→FeO→Fe). At 1373 K, thermodynamic equilibrium favored near-complete metallic iron formation across all systems despite reductant-dependent kinetic variations observed at lower temperatures.

**Figure 7:**
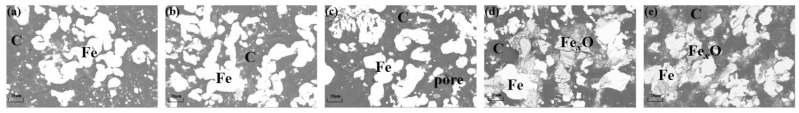
Microstructural morphology of carbon-bearing briquettes calcined at 1273 K using different reducing agents: (**a**) wood char; (**b**) bamboo char; (**c**) semi-coke; (**d**) anthracite; (**e**) coke powder.

**Figure 8:**
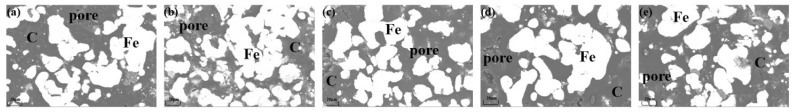
Microstructural morphology of carbon-bearing briquettes calcined at 1373 K using different reducing agents: (**a**) wood char; (**b**) bamboo char; (**c**) semi-coke; (**d**) anthracite; (**e**) coke powder.

Following calcination at 1273 K and 1373 K for 30 min, metallization rates of carbon-bearing briquettes with various reductants (wood char, bamboo char, coke powder, semi-coke, anthracite) were determined via TFe/MFe analysis, and the results are summarized in Table 3 and Table 4. Concurrent microstructural evolution was characterized through SEM analysis of briquettes reduced under identical conditions (1273/1373 K, 30 min), with the corresponding micrographs presented in Figure 7 and Figure 8, respectively.

Following calcination at 1273 K, metallization rates reached 73.08% and 75.67% for coke powder and anthracite-based briquettes, respectively. Phase analysis confirms significant metallic iron formation at this temperature. Semi-coke formulations exhibited enhanced reduction (78.45% metallization), while bamboo char briquettes achieved optimal performance (88.60% metallization), which is the highest among all reductants. This superior reducibility originates from the bamboo char’s developed surface porosity and high specific surface area, which facilitates accelerated fixed carbon gasification. The consequent CO-rich atmosphere enhances iron oxide reduction kinetics through improved gas–solid reaction dynamics, thereby promoting briquette metallization.

Following calcination at 1373 K, metallization rates increased universally across all reductant systems. Among them, bamboo char carbon-bearing briquettes had the highest metallization rate, reaching 90.16%. This was mainly due to the good reactivity of bamboo char, its high fixed carbon content, and its well-developed pores, which provided sufficient atmospheric conditions for the reduction of carbon-bearing briquettes after the temperature rose. The second highest was wood char-containing carbon briquettes, with a metallization rate of 87.00%. The metallization rate test results for coke powder, anthracite, and semi-coke were consistent with their reactivity rankings, and the metallization rates were all above 80.00%.

### 3.4. Reaction Mechanism of the Biochar Reducing Agent

**Figure 9:**
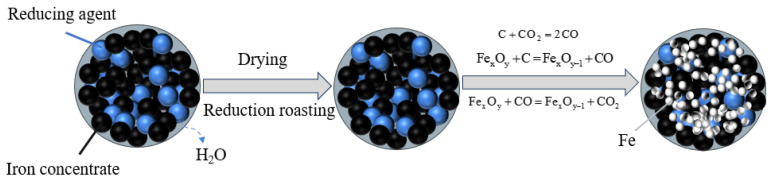
Schematic diagram of the process of reducing iron ore with biochar as a reducing agent.

At elevated temperatures (>1273 K), biochar reductants (wood char and bamboo char) exhibit enhanced reducing capacity, driving the progressive reduction of iron oxides to metallic iron. This transformation encompasses three synergistic pathways: direct solid–solid reduction, indirect gas–solid reduction, and carbon gasification, as shown in the corresponding reaction Equations (3)–(5). Figure 9 schematically illustrates this multi-mechanistic reduction process, highlighting biochar’s role in facilitating iron oxide conversion through interconnected reaction channels.

(3) $${\mathrm{Fe}}_{x}O_{y}+{C=\mathrm{Fe}}_{x}O_{y-1}+\mathrm{CO}$$

(4) $${\mathrm{Fe}}_{x}O_{y}+{\mathrm{CO}=\mathrm{Fe}}_{x}O_{y-1}{+\mathrm{CO}}_{2}$$

(5) $${C+\mathrm{CO}}_{2}=2\mathrm{CO}$$

where Fe*_x_*O*_y_* represents Fe_2_O_3_, Fe_3_O_4_, and FeO, respectively; Fe*_x_*O*_y_*_−1_ represents Fe_3_O_4_, FeO, and Fe, respectively.

There is always a significant positive correlation between the external surface area of carbonaceous materials and the amount of iron reduction [28]. From the SEM images of wood char and bamboo char, it can be seen that wood char and bamboo char have a high specific surface area and good pore structure. This unique structural characteristic allows CO to diffuse more quickly to the iron oxide interface, strengthening gas–solid mass transfer, enabling the reaction system to utilize carbon resources more efficiently, and thereby promoting rapid reduction reactions [29].

During the initial reduction stage, carbon-bearing briquettes undergo direct solid–solid reduction where biochar-derived carbon establishes intimate interfacial contact with iron oxide particles, mediating the reduction process. As the temperature rises, the direct reduction reaction between carbon and iron oxide becomes significantly enhanced at the interface [30]. This synergistic mechanism not only accelerates the direct contact and reaction between carbon and iron oxide but also further promotes the carbon gasification reaction and indirect reduction reaction. When the temperature rises further, the CO generated by the carbon gasification reaction participates in the reduction process of iron ore, and the synergistic effect of the solid–solid reaction and the gas–solid reaction significantly increases the reduction rate, facilitating rapid metallic iron nucleation. Consequently, biochar reducing agents (wood/bamboo char) achieve higher metallization rates than semi-coke, anthracite, or coke powder systems.

## 4. Conclusions

This study systematically compared biochar with conventional fossil-based reductants in the reduction of carbon-bearing briquettes, providing both mechanistic insights and practical evaluation. The findings highlight not only the fundamental differences in reactivity and metallization behavior but also the potential role of biochar in advancing low-carbon ironmaking. The following key conclusions can be drawn:

(1) Biochar reducing agents have better reactivity than anthracite and coke powder. At 1173 K, the conversion rates of wood char, bamboo char, and semi-coke are close to 100%, while anthracite and coke powder still have a weight loss rate of less than 100% at 1273 K. This is because biochar has a loose structure, large specific surface area, high reactivity, and is easy to gasify; anthracite and coke powder have a dense structure, small specific surface area, low reactivity, and require higher temperatures for gasification.

(2) An appropriate C/O molar ratio can ensure that there is sufficient carbon in the reaction system to participate in the reduction. If the C/O molar ratio is too low, reduction will be hindered and the metallization rate will be low; if it is too high, the reduction rate will decrease and costs will increase. Therefore, C/O = 0.8 was selected for the preparation of carbon-bearing briquettes. At 873 K, the carbon-bearing briquettes of different reducing agents had high strength, with compressive strength exceeding 800 N, but reduction had not yet begun. When the temperature rose to 1273 K, the compressive strength of the briquettes reached its lowest point. At 1373 K, due to the production of a large amount of metallic iron, the strength of the briquettes increased, and the metallization rate also increased.

(3) Bamboo char achieved peak metallization performance (90.16%) in carbon-bearing briquettes, attributable to its synergistic combination of enhanced gasification kinetics and elevated fixed carbon content (>85%), which generated highly reducing atmospheres at elevated temperatures. Wood char demonstrated secondary efficacy, while metallization rates for coke powder, anthracite, and semi-coke aligned with their established reactivity hierarchy. The macroporous architectures of wood and bamboo chars facilitated the accelerated diffusion of CO to iron oxide interfaces, strengthening gas–solid mass transfer. Progressive heating enhanced direct carbon–iron oxide reduction while concurrently promoting the Boudouard reaction and indirect reduction pathways. This multi-mechanistic coupling increased reduction kinetics relative to conventional reductants, optimizing metallic iron nucleation. Consequently, biochar reducing agents (wood/bamboo char) achieve higher metallization rates than semi-coke, anthracite, or coke powder systems.

(4) From a low-carbon perspective, the application value of biochar in carbon-bearing briquette reduction depends on two key premises: first, the biomass consumed for biochar production must be replenished by planting equivalent fast-growing plants to maintain the balance of the fast carbon cycle, ensuring “conditionally carbon-free” substitution of fossil carbon; second, the energy used in biochar preparation should prioritize low-carbon sources to reduce carbon emissions from the production link. This study confirms that biochar can improve the reduction efficiency of carbon-bearing briquettes, but future research should further quantify the full-life-cycle carbon footprint of biochar (from biomass cultivation to biochar preparation and application) to more accurately evaluate its contribution to the low-carbon transformation of the iron and steel industry.

## Figures and Tables

**Table 1:** Main chemical components of iron concentrate/%.

TFe	FeO	SiO_2_	MgO	Al_2_O_3_	CaO	MnO	Na_2_O	Others
70.84	22.76	1.18	1.01	0.58	0.36	0.25	0.11	2.91

**Table 2:** Physical and chemical properties of different reducing agents.

Sample	Proximate Analysis (wt.%)			Ultimate Analysis (wt.%)				
	V_d_	A_d_	FC_d_ *	C_d_	H_d_	O_d_ *	N_d_	S_d_
Wood char	5.77	8.57	85.66	86.49	0.39	4.07	0.36	0.12
Bamboo char	7.08	2.76	90.16	87.39	2.12	6.96	0.77	-
Semi-coke	13.71	12.07	74.22	75.66	1.74	9.18	0.87	0.48
Coke powder	1.24	12.86	85.90	84.08	0.26	0.90	1.08	0.82
Anthracite	6.79	7.94	85.27	86.37	1.61	3.02	0.75	0.41

Note: V: volatile matter; A: ash; FC: fixed carbon, d: air dried basis; *: by difference.

**Table 3:** Metallization rate of carbon-bearing briquettes at 1273 K using different reducing agents, %.

Sample	MFe	TFe	M
Wood char	60.22	75.54	79.72
Bamboo char	72.06	81.33	88.60
Semi-coke	60.89	77.62	78.45
Anthracite	55.95	73.94	75.67
Coke powder	47.85	65.48	73.08

**Table 4:** Metallization rate of carbon-bearing briquettes at 1373K using different reducing agents, %.

Sample	MFe	TFe	M
Wood char	70.45	80.98	87.00
Bamboo char	76.58	84.94	90.16
Semi-coke	67.27	79.53	84.58
Anthracite	66.49	78.14	85.09
Coke powder	64.20	78.33	81.96

## Data Availability

The original contributions presented in this study are included in the article. Further inquiries can be directed to the corresponding authors.

## Abbreviations

The following abbreviations are used in this manuscript:

C/O	carbon-to-oxygen
M	metallization rate
